# Coupled Intrinsic Connectivity Distribution Analysis: A Method for Exploratory Connectivity Analysis of Paired fMRI Data

**DOI:** 10.1371/journal.pone.0093544

**Published:** 2014-03-27

**Authors:** Dustin Scheinost, Xilin Shen, Emily Finn, Rajita Sinha, R. Todd Constable, Xenophon Papademetris

**Affiliations:** 1 Department of Biomedical Engineering, Yale University, New Haven, Connecticut, United States of America; 2 Department of Diagnostic Radiology, Yale University, New Haven, Connecticut, United States of America; 3 Interdepartmental Neuroscience Program, Yale University, New Haven, Connecticut, United States of America; 4 Department of Psychiatry, Yale University, New Haven, Connecticut, United States of America; 5 Department of Neurosurgery, Yale University, New Haven, Connecticut, United States of America; Brainnetome Center, & National Laboratory of Pattern Recognition, China

## Abstract

We present a novel voxel-based connectivity approach for paired functional magnetic resonance imaging (fMRI) data collected under two different conditions labeled the Coupled Intrinsic Connectivity Distribution (coupled-ICD). Our proposed method jointly models both conditions to incorporate additional paired information into the connectivity metric. Voxel-based connectivity holds promise as a clinical tool to characterize a wide range of neurological and psychiatric diseases, and monitor their treatment. As such, examining paired connectivity data such as scans acquired pre- and post-intervention is an important application for connectivity methodologically. When presented with data from paired conditions, conventional voxel-based methods analyze each condition separately. However, summarizing each connection separately can misrepresent patterns of changes in connectivity. We show that commonly used methods can underestimate functional changes and subsequently introduce and evaluate our solution to this problem, the coupled-ICD metric, using two studies: 1) healthy controls scanned awake and under anesthesia, and 2) cocaine-dependent subjects and healthy controls scanned while being presented with relaxing or drug-related imagery cues. The coupled-ICD approach detected differences between paired conditions in similar brain regions as the conventional approaches while also revealing additional changes in regions not identified using conventional voxel-based connectivity analyses. Follow-up seed-based analyses on data independent from the voxel-based results also showed connectivity differences between conditions in regions detected by coupled-ICD. This approach of jointly analyzing paired resting-state scans provides a new and important tool with many applications for clinical and basic neuroscience research.

## Introduction

Functional connectivity holds promise as a tool to detect abnormal brain organization in clinical populations, and to monitor functional changes in response to treatment [1]. When monitoring treatments, exploratory analysis at a whole-brain level with few or no *a priori* assumptions is often desired so as to maximize the likelihood of observing any and all changes in function (including potentially adverse changes). Conventional task-based functional magnetic resonance imaging (fMRI) is often inadequate for whole-brain functional monitoring because only a few regions reliably activate above baseline for any given task, and interactions between task performance and conditions can confound serial measurements. Many functional connectivity approaches such as [2]–[4] rely on regions of interests (ROIs) to characterize the effects of drugs/treatment on connectivity. However, these approaches can only examine regions that are *a priori* hypothesized to show changes, and so potentially important regions may not be examined due to limited *a priori* knowledge. As such, whole-brain analysis methods designed for paired fMRI data such as scans acquired pre- and post-intervention is an important area of development for connectivity methodology.

Voxel-based measures of connectivity are emerging tools for monitoring functional changes between conditions in whole-brain studies [5]–[9]. These methods can be viewed as a generalization of ROI-based approaches where each voxel is an ROI. Thus, every region is directly examined. Nevertheless, using each voxel as an ROI results in large connectivity matrices (with more than 10,000×10,000 correlations at typical fMRI resolutions) that are difficult to interpret and are problematic for statistical inferences. To simplify, voxel-based metrics essentially work as compression mechanisms, reducing all information about the connections to a voxel into a few summary statistics. For example, the network theory measure *degree* reduces a given row of the connectivity matrix to a single number by counting the number of connections above a correlation threshold [5], [6], [9], or by averaging correlations [5], [8]. However, this compression comes at a price as spatial information about specific connections to that voxel is lost.

This loss of information can cause additional problems in the case of paired conditions such as pre- and post- treatment. Here, the standard approach is to compute voxel–based metrics separately for each condition and then perform statistical analysis to compare the two. However, we observe that this approach can be suboptimal as the compression into a summary statistic is performed twice (once for each condition). If thresholds are involved in creating this summary statistic, the difference in the summary statistic is not always the same as the summary statistic of a difference. Hence, in addition to losing spatial information as stated above, information about how each connection changes due to the treatment can be lost with current approaches.

In this work, we propose a method to take advantage of additional information held by paired conditions where within-subject differences across conditions are first computed and then a single summary measure can be calculated for these differences. The coupled Intrinsic Connectivity Distribution (coupled-ICD) presented here extends the recently developed Intrinsic Connectivity Distribution (ICD) method [7] and jointly analyzes each of the paired conditions at the voxel level. ICD, and by extension coupled-ICD, represent a generalization of the network-theory measure degree [5], [6], [10] and model the change in degree as the threshold used to calculate degree is increased. The ICD approach was developed in order to eschew the need to choose an arbitrary threshold to determine if two voxels are connected or not — a weakness of previous approaches. The coupled-ICD approach retains this advantage of examining the entire connectivity spectrum without requiring the selection of an arbitrary threshold to determine connectivity.

To assess our coupled-ICD measure, we used two data sets of paired scans. The first consisted of healthy controls scanned awake and under anesthesia, while the second consisted of cocaine-dependent subjects and healthy controls scanned while presented with relaxing and drug-related imagery cues. We show that coupled-ICD has higher sensitivity than conventional approaches for detecting differences between conditions. Finally, on a separate, independent sub-sample of our data, we show that regions detected by coupled-ICD are predictive of seed-based differences in connectivity.

## Methods

### Ethics Statement

Data were from studies performed at Yale University School of Medicine, New Haven, CT. All protocols were reviewed and approved by Human Research Protection Program at Yale University. Written informed consent was obtained. All scans were obtained and analyzed at Yale University.

### Theory

Voxel-based measures of functional connectivity [6]–[9], [11] aim to reduce large amounts of information about connectivity to a voxel into a much smaller set of summary statistics. Typically, these methods have their roots in graph theory [10], in which the brain is treated as a “graph,” or network, and each voxel represents a node in this graph. These nodes (or voxels) are connected to each other by “edges,” or connections, based on the similarity of their timecourses.

Measures of node centrality such as *degree* are the primary metrics used for voxel-based connectivity. These measures can be calculated from the distribution of correlations for any voxel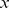. First, 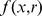 is defined as the distribution of the correlations 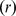 for the timecourse at voxel 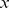 to the timecourse at every other voxel in the brain and can be estimated by computing the histogram of these correlations. Degree, based on a binary graph, can be estimated as the integral of this distribution from any threshold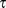 to 1, or 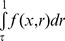. Weighted degree measures such as weighted Global Brain Connectivity (wGBC) [8] can be estimated as the mean of this distribution or a distribution of transformed correlations. In contrast, ICD models the corresponding survival function to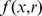. Each point on the survival function is simply degree, based on a binary graph, evaluated at that particular threshold 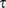. The ICD approach is to parameterize the change in a voxel's degree as the threshold used to determine if two voxels are connected is increased. Previously [7], we showed that a stretched exponential decay with unknown variance parameter 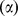 and shape parameter 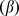 was sufficient to model this survival function. Modeling the survival function with a stretched exponential is equivalent to modeling the underlying distribution as a Weibull distribution: 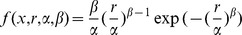, where 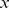
is the spatial location of a voxel, 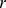 is a correlation between two timecourses,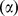 is the variance parameter, and 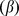 is the shape parameter. Thus, ICD models the variance of the distribution of correlations between a voxel and every other voxel in the brain. No thresholds are needed to estimate the variance or model the distribution. 

**Figure 1:**
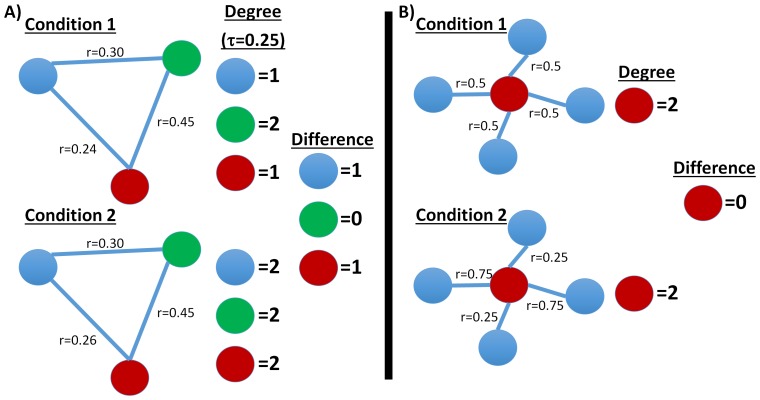
Examples of how conventional approaches that separately summarize each condition of a pair could misrepresent patterns of changes in connectivity. A) When a binary graph is used, changes in correlation near the threshold value (threshold 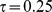) can lead to an over/under-estimation of connectivity changes. In this example, one edge increases its correlation by 0.02 in between conditions 1 and 2, which leads an increase in degree for condition 2. However, this increase in correlation and degree is likely not meaningful. B) When a weighted graph is used, increases and decreases in connectivity between conditions could cancel each other out. In this example, half of a node's edges increase their correlation while half of its edges decrease their correlation in condition 2 compared to condition 1. When all edges are averaged together, no change between the conditions is detected, despite that a change is clearly present.

We observe that the difference between the summary statistics (degree, wGBC, or ICD) of two graphs may not be the same as a summary of the difference between the graphs. Two examples are shown in Figure 1A and 1B where comparing summary statistics of two graphs can potentially either overestimate or underestimate a change in paired conditions. The first example uses degree based on a binary graph where one connection changes a small amount going from just below the threshold (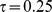) to just above the threshold. This change causes degree for two nodes to increase in the second condition even though the change in correlation was relatively small. The second example uses degree based on a weighted graph where, in the second condition, an equal amount of connections increase in strength and decrease in strength compared to the first condition. This changes results in no difference in degree between the two conditions for the selected node. Given that the standard approach is to compute voxel–based metrics separately for each condition and then perform statistical analysis to compare these two metrics (see for example, [12]), we hypothesize that summarizing the difference between two graphs may be a more powerful alternative approach.

**Figure 2:**
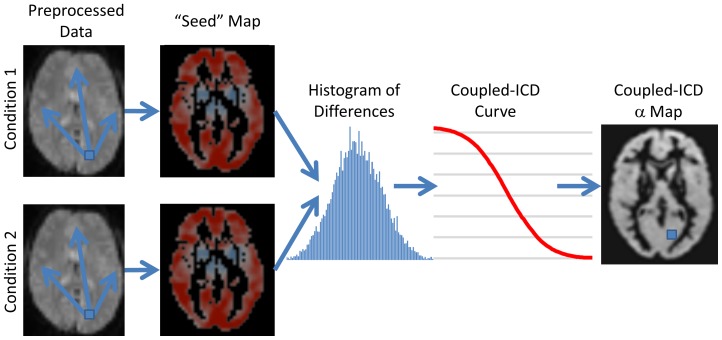
Flow chart describing coupled-ICD. For data consisting of paired conditions, coupled-ICD jointly analyzes both conditions and then creates a summary of the difference in connectivity between conditions for each voxel. First, a “seed” connectivity map is created for a voxel (shown as the blue square through the flow chart) in each condition. The resulting “seed” maps are then subtracted and a histogram of the differences is computed. The survival function of the distribution of the difference (labeled as coupled-ICD curve) is calculated and modeled with a stretched exponential. This process is repeated for each voxel in the gray matter. The final output is an image where each voxel represents a summary of the difference between two “seed” maps using that voxel as the seed region.

The present approach, coupled-ICD, extends conventional voxel-based connectivity in a critical way: the graph summarized by coupled-ICD is a graph defined by *differences* in connectivity, not connectivity itself. As such, coupled-ICD takes advantage of the paired nature of the data by explicitly comparing the strength of the same edge (the strength of an edge is the correlation between the time courses of a pair of voxels) two conditions. Given a set of paired data, coupled-ICD can be computed by repeatedly calculating conventional seed connectivity maps treating each voxel as a seed, and summarizing the difference between the seed maps for each condition (see Figure 2). First, for any voxel 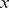, the correlation between the timecourse at voxel 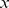 to the timecourse at every other voxel in the gray matter is calculated for each condition in the paired data. These correlation maps are then subtracted from one another. Coupled-ICD then summarizes this map of differences in the same way that ICD (or *degree*) summarizes a map of connections to a voxel. First, for each voxel, a distribution of these differences is estimated with a histogram. Second, this distribution is modeled as a Wiebull distribution, which corresponds to modeling the survival function of the histogram as a stretched exponential.

The coupled-ICD approach can be used to model increases in connectivity, decreases in connectivity, or the magnitude (absolute value) of the changes in connectivity. In all cases, a larger α parameter indicates that the distribution has a larger variance, thus indicating that a larger number of connections exhibit a strong change in correlations between the two conditions. Group comparisons can be performed by comparing the α parameters using standard statistical testing.

### Subjects

Coupled-ICD can be used as an exploratory tool to help select regions that are most likely to show significant differences upon further analysis in the cases of rs-fMRI data arising from two different conditions. We evaluate this aspect of coupled-ICD using data set 1. Additionally, if two separate groups have paired condition data, it is possible to use coupled-ICD directly to estimate regions of statistically significant connectivity differences. We evaluate this aspect of coupled-ICD using data set 2.

#### Data Set 1

The first data set consisted of 14 healthy subjects and aimed to examine the influence of anesthesia on intrinsic functional connectivity. Each subject was scanned at rest for nine 6-minute runs. In the first three (pre-anesthesia) and last three (post-anesthesia) scans, pure oxygen was administrated to the subjects. In the second three (anesthesia) scans, sevoflurane (0.5 MAC) was added to the pure oxygen. A 10-minute gap was introduced between experimental sessions to allow for end-tidal sevoflurane concentration to reach a steady-state and to allow for anesthetic washout, respectively. During the entire study, subjects were asked to lie in the scanner with their eyes closed and to refrain from performing any goal-oriented mental activity. Only the first two conditions were analyzed. Full details about this data set and previous results can be found elsewhere [13].

#### Data Set 2

The second data set consisted of 28 cocaine-dependent subjects and 38 healthy control subjects and aimed to examine the influence of cue state and diagnostic group on brain activity. Subjects performed four fMRI scans while listening to imagery scripts of either neutral relaxing cues or drug-related cues (two scans of each). These imagery scripts were custom tailored to each subject and no script was presented twice. Each scan lasted 5 minutes and consisted of three blocks including a 1.5-minute quiet baseline period, a 2.5-minute imagery period, and a 1-minute quiet recovery period. For connectivity analysis, only the large, continuous 2.5-minute imagery period was used. As only the imagery period was analyzed, task effects were not regressed in the presented results. Results with task effects regressed are visually similar and, for simplicity, not presented. Complete details about the sample and imagery script design can be found elsewhere [14], [15]. This data set was added to the study because it consists of two groups with multiple conditions, which allows for the comparison of coupled-ICD parameters using standard statistical testing (see the Statistical Analysis section below).

### Imaging Parameters

#### Data Set 1

Imaging was performed using a 3T Siemens (Erlangen, Germany) Trio MR system. After a first localizing scan, 33 axial slices (slice thickness 4 mm, no gap, FoV = 256 mm, matrix size 256×256) parallel to the AC-PC line were acquired using a T1-weighted sequence (TR = 300 ms, TE = 2.43 ms, FoV = 256 mm, matrix size 256×256, flip angle 60°). Functional imaging volumes were collected in the same slice position as the preceding T1-weighted data. For each experimental condition, three functional runs were acquired using a T2*-sensitive gradient-recalled, single-shot echo-planar imaging pulse sequence (TR = 2 s, TE = 31 ms, FoV = 256 mm, flip angle 90°, matrix size 64×64). Each volume consisted of 33 slices parallel to the bicommissural plane (slice thickness 4 mm, no gap), and each functional run comprised 210 volumes. High-resolution anatomical images were acquired using a T1-weighted sagittal gradient-echo (MPRAGE) sequence (176 contiguous sagittal slices, slice thickness 1 mm, matrix size 256×256, FoV = 256 mm TR = 2530 ms, TE = 3.34 ms, flip angle = 7°).

#### Data Set 2

Imaging was performed using a 3T Siemens (Erlangen, Germany) Trio MR system. After a first localizing scan, 32 axial slices (slice thickness 4 mm, no gap, FoV = 220 mm, matrix size 256×256) parallel to the AC-PC line were acquired using a T1-weighted sequence (TR = 300 ms, TE = 2.5 ms, FoV = 220 mm, matrix size 256×256, flip angle 60°). Functional imaging volumes were collected in the same slice position as the preceding T1-weighted data. For each experimental condition, three functional runs were acquired using a T2*-sensitive gradient-recalled, single-shot echo-planar imaging pulse sequence (TR = 2 s, TE = 25 ms, FoV = 220 mm, flip angle 85°, matrix size 64×64). Each volume consisted of 32 slices parallel to the bicommissural plane (slice thickness 4 mm, no gap), and each functional run comprised 180 volumes. High-resolution anatomical images were acquired using a T1-weighted sagittal gradient-echo (MPRAGE) sequence (176 contiguous sagittal slices, slice thickness 1 mm, matrix size 256×256, FoV = 256 mm TR = 2530 ms, TE = 3.66 ms, flip angle = 7°).

### Preprocessing

Images were slice-time corrected using sinc interpolation and motion corrected using SPM5 (http://www.fil.ion.ucl.ac.uk/spm/software/spm5/). All further analysis was performed using BioImage Suite [16]. Several covariates of no interest were regressed from the data including linear and quadratic drift, six rigid-body motion parameters, mean cerebrospinal fluid (CSF) signal, and mean white matter signal. The global signal was not removed. The white matter and CSF areas were defined on a template brain [17], eroded to ensure only white matter or CSF signal would be included, and warped to individual subject space using a series of transformations described below. Finally, the data were temporally smoothed with a zero mean unit variance Gaussian filter (approximate cutoff frequency = 0.12 Hz). For each subject, connectivity was estimated for each voxel in each subject's individual space. First, a gray-matter mask was applied to the data so that only voxels in the gray matter were used in the calculation. The gray-matter mask was defined on a template brain [17], dilated to ensure full coverage of the gray matter, and warped to individual subject space using a series of transformations described below.

#### Common space registration

To facilitate group statistics, all single-subject results (ICD wGBC, coupled-ICD, and seed maps) were first warped to a common template space through the concatenation of a series of linear and non-linear registrations and spatially smoothed with a 6 mm Gaussian filter. The functional series were linearly registered to the T1 axial-oblique (2D anatomical) image. The 2D anatomical image was linearly registered to the MPRAGE (3D anatomical) image. The 3D anatomical image was non-linearly registered to the template brain. All transformation pairs were calculated independently and combined into a single transform between single-subject space and common space, reducing interpolation error. All transformations were estimated using the intensity-only component of the method implemented in BioImage Suite [18].

### Functional connectivity estimation

#### Division of data sets

As coupled-ICD (along with most voxel-based metrics) compresses spatial information about changes in connectivity to a single parameter for any voxel, we performed standard seed-based analysis with seeds derived from regions detected by coupled-ICD to explore which specific connections are responsible for the change in connectivity. This follow-up seed analysis provides additional, though not direct, evidence that differences detected reflect “true” changes in connectivity. In order to show that regions detected by coupled-ICD are predictive of changes with seed-based methods, the voxel-based analysis and follow-up seed-based analysis were performed on separate, independent subsets of the data.

All data was split into two groups for each data set. One group was used for the voxel-based analysis. The other group was used for follow-up seed analysis on regions detected by coupled-ICD. For data set 1, given the limited sample of subjects (n = 14) but large amount of imaging data per condition (approximately 15 minutes), the data set was split into two groups by runs. For each subject, two runs for each condition were randomly chosen for voxel-based analysis. The remaining run was left out for follow-up seed analysis. In contrast, for data set 2, which had a larger sample of subjects (38 controls and 28 cocaine-dependent individuals) but less imaging data per condition (approximately 5 minutes), the data was split into two groups of subjects. Fourteen cocaine-dependent subjects and 19 healthy controls were randomly chosen for voxel-based analysis. The remaining subjects were used for seed-based analysis. Runs were temporally concatenated when there were multiple runs for a condition. Additionally the data reserved for seed analysis was also used to replicate the primary coupled-ICD analysis. This data is presented in supplemental material (see also Figure S1, S2, S3, S4, S5, S6).

#### ICD and wGBC estimation

The timecourse for voxel 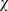 was correlated with the timecourse for every other voxel in the gray matter. For each voxel, a distribution of connection strength was estimated for the positive correlation coefficients using a 100-bin histogram. ICD was used to model this distribution as described in [7]. First, the histogram was converted to the corresponding survival function and this survival function was modeled with a stretched exponential. This results in two summary statistics for each voxel reflecting that voxel's connectivity to the rest of the brain; the alpha parameter was used in the group comparisons. The wGBC maps were estimated as the mean of this histogram (i.e., the sum of all correlations to a voxel divided by the total number of correlations). Both the ICD and the wGBC maps for each paired condition were then subtracted from each other, resulting in a single map per subject describing the difference in connectivity between conditions. ICD and wGBC were estimated in single subject space and then warped to common space.

#### Matrix connectivity

As coupled-ICD summarizes the differences in correlations (edges) rather than (node-based) graph theory measures, we included a non-voxel-based approach that divides the brain into 278 distinct regions and interrogates connectivity between these regions [19]. For each subject, the Shen functional atlas [20], [21] (available for download at http://www.nitrc.org/projects/bioimagesuite/) was warped to individual space and the pairwise correlation coefficient between the timecourses of each possible pair of nodes was computed. The correlations were transformed to Z scores with the Fisher transformation, resulting in a 278×278 symmetric connectivity matrix for each subject. These matrix were then used in second level group analysis.

#### Coupled-ICD analysis

Similar to the ICD and wGBC estimations described above, the timecourse for voxel 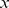 was correlated with the timecourse for every other voxel in the gray matter. As coupled-ICD operates on paired data, this process was performed on both conditions resulting in two seed connectivity maps with voxel 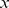 as the seed. These maps are then subtracted. A distribution of the differences in connection strength was estimated for the absolute value of the differences using a 200-bin histogram. A larger number of bins was used to keep the bin width the same as the ICD analysis while accommodating the wider range of possible values (difference in correlations has a theoretical range of [−2,2] while correlation has a range of [−1,1]). We chose to model increases and decreases in connectivity separately for data set 1. For this case, the distribution of differences in correlation was split into two halves and the positive and negative halves of the distribution were converted to separate survival curves for coupled-ICD analysis. For data set 2, we chose to model the overall change in connectivity to highlight regions of the brain that showed large differences between the two conditions. The absolute value of the distribution of differences in correlation was taken and converted to a survival function. As described above, these survival curves were modeled with a stretched exponential reducing the functional connectivity metric for a voxel under two conditions into two summary statistics (the alpha and beta parameters characterizing the survival curve). The alpha parameter was used for group comparisons and represents a summary of the difference in a voxel's connectivity between two conditions. As both conditions were acquired during the same imaging session, the two conditions for each subject were already registered. Thus, coupled-ICD was estimated in single subject space and then warped to common space.

#### Seed-based connectivity analysis

Follow-up seed-based analyses (similar to [6], [22], [23]) were performed on sample regions detected using coupled-ICD. For data set 1, two seeds were placed in the right parietal lobe (BA 39) and left lateral prefrontal cortex (BA 10). For data set 2, a seed was placed in left putamen based on voxels showing significant differences (p<0.05 corrected) between controls and cocaine-dependent subjects.

All seeds were defined on the MNI reference brain and transformed back (via the inverse of the transforms described below) into individual subject space. The timecourse of the reference region in a given subject was then computed as the average timecourse across all voxels in the reference region. This timecourse was correlated with the timecourse for every other voxel in the gray matter to create a map of r-values, reflecting ROI-to-whole-brain connectivity. These r-values were transformed to z-values using Fisher's transform, yielding one map for each subject representing the strength of correlation to the seed region.

#### Statistical analysis

Highly connected regions for single-group results were detected using a modified version of the Top Percent method [8]. This method was developed for connectivity analysis of a single group where the null hypothesis is not obvious, and has been shown to be a reliable surrogate for control for false positives. For this method, the group average was divided by the group standard deviation for each voxel resulting in a measure of within-group effect size. Any voxels in the top 15 percent of effect size were considered highly connected. A cluster threshold of 50 voxels was used for visualization. This measure highlights voxels that are both highly connected to the rest of the brain and highly consistent among the sample population. This method was used for the coupled-ICD results for data set 1.

Between-group differences for coupled-ICD results from data set 2, conventional measures, and matrix data were identified using t-tests. As the ICD (and coupled-ICD) parameters are found via regression analysis, we justify the use of parametric testing because the errors from the regression analysis are likely normally distributed and independent give reasonably large sample size used to estimate these parameters. Significance was assessed at a p<0.05 level corrected for multiple comparisons. Multiple comparisons were accounted for using AFNI's AlphaSim program for imaging data and false discovery rate for the matrix data. All results were also localized in terms of the Brodmann areas (BA) identified using BioImage Suite's digital Brodmann atlas.

#### Motion analysis

As group differences in motion have been shown to confound functional connectivity results, average frame-to-frame displacement was calculated for each group [24]. There was no significant group difference (p = 0.54) in motion for data set 1 between the awake and anesthesia runs. There were no significant group differences in motion between healthy controls and cocaine-dependent subjects for data set 2 for either the relaxing imagery (p = 0.57) or the drug-related imagery (p = 0.45) conditions.

#### Group comparisons:

In the Results section, we presented two examples of the coupled-ICD approach. For data set 1, we wished to explore the effect of anesthesia on brain connectivity and wanted to characterize all changes in connectivity due to condition. We decided to model the increases and decreases separately in order capture the directional main effects of anesthesia. This example highlights how coupled-ICD can be used to define reduce the search space to focal seed regions for further independent analysis. For data set 2, we expected that both drug dependent individuals and control subjects would show connectivity changes as a function of condition. However, we were not explicitly interested in this change but rather how drug dependent subjects differed from controls in their response to drug cues. Thus our primary interest is the interaction, not directional main effects. As such, we examined the absolute value to detect any changes in connectivity regardless of direction. This is similar to looking the F-statistic to detect significant interaction. This example highlights how coupled-ICD can be used as a direct statistical method for quantifying group connectivity differences.

## Results

### Exploration of increased and decreased connectivity under anesthesia

**Figure 3:**
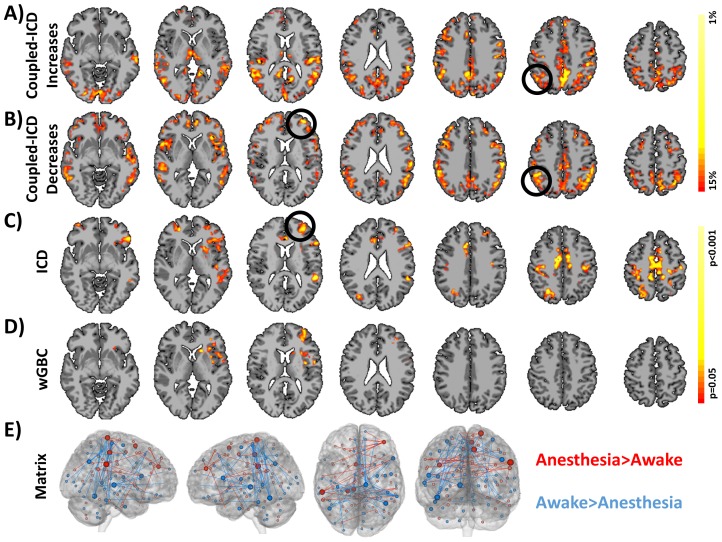
Comparison of coupled-ICD and conventional approaches for detecting connectivity changes due to anesthesia. Regions of large A) increased connectivity and B) decreased connectivity under anesthesia detected by coupled-ICD and thresholded using the Top Percent method. For some regions, coupled-ICD was able to detect regions with both increased *and* decreased connectivity. One of these areas (the right parietal lobe; black circle) was used for further analysis. Regions of significant change in connectivity detected by conventional voxel-based approaches are shown in C) ICD and D) wGBC. While a general correspondence was observed between all methods, the coupled-ICD results suggest a decrease in connectivity for the left frontal lobe (black circle) while the conventional approaches suggest an increase in connectivity. This region was selected for further analysis. While the conventional voxel-based approaches, C) ICD and D) wGBC, suggested more focal changes in connectivity, E) matrix connectivity and coupled-ICD suggest more widespread changes due to anesthesia. Only edges that were significantly difference at p<0.05 with FDR correction are shown. The size of the node is proportional to the number of significantly different edges touching that node such that a larger node has more significantly different edges.

Using data set 1, we explored how coupled-ICD detected changes in connectivity due to anesthesia. Specifically, we explored which regions showed the largest increases and decreases in connectivity when the anesthesia condition was compared to the awake condition by observing which regions constituted the top 15 perfect of the effect size of the coupled-ICD alpha parameter. This approach effectively creates “hub” maps of increased and decreased connectivity (shown in Figure 3A and 3B, respectively). Coupled-ICD detected several regions of large increased connectivity during the anesthetized condition relative to the awake condition. These regions include the supplementary motor area (SMA)/anterior cingulate cortex (ACC), lateral parietal lobe, posterior cingulate cortex (PCC)/ precuneus, and visual cortex. Sparse increases were also found in the lateral frontal lobe. Regions of large decreased connectivity were detected in the dorsal lateral prefrontal cortex, insular cortex, medial prefrontal cortex, anterior and posterior cingulate cortex, and lateral parietal lobe.

Notably, certain areas showed both large increases and decreases in connectivity as measured by coupled-ICD. These areas were mostly in the PCC/precuneus and lateral parietal lobe. Standard analysis with either wGBC or ICD (shown in Figure 3C and 3D, respectively) —where each condition is summarized separately—can only specify if an area shows either increased *or* decreased connectivity and cannot specify if an area shows both increased *and* decreased connectivity. While the coupled-ICD and the conventional approaches (wGBC, ICD, and matrix connectivity) detect similar regions of altered connectivity (albeit with different overlay methods), coupled-ICD is the only voxel-based method that can highlight regions where connectivity changes in *both* directions.

**Figure 4:**
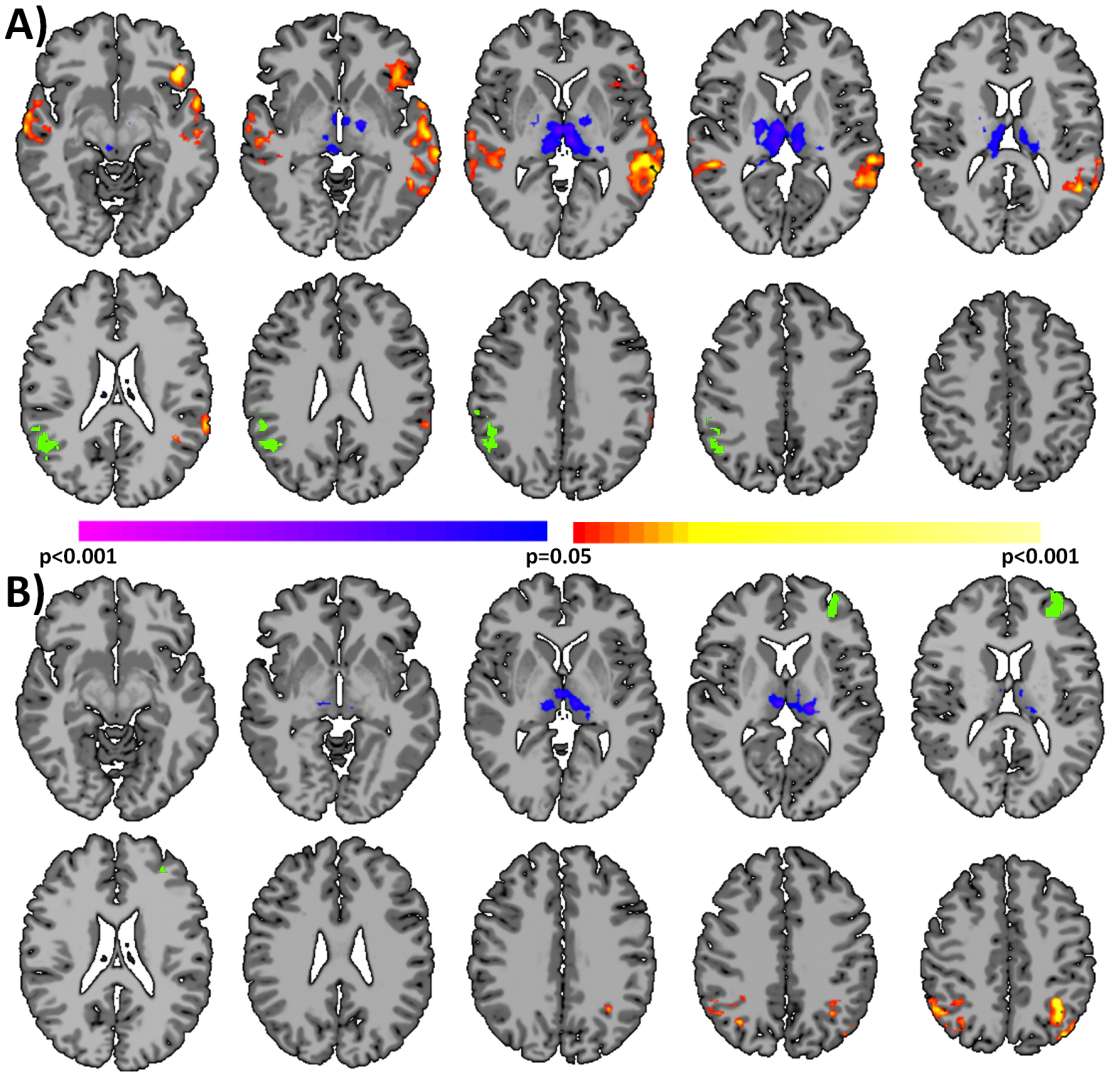
Seed-based connectivity results using seeds detected by coupled-ICD. A) Several regions of the brain displayed evidence for both increased and decreased connectivity during the anesthesia state as detected by coupled-ICD. A follow-up seed-based analysis on independent data for one of these regions (the right parietal lobe; green region) revealed both significant (p<0.05 corrected) increases and decreases in connectivity to this region, echoing the coupled-ICD results. B) For some regions of the brain, coupled-ICD and conventional approaches showed seemingly conflicting results, with coupled-ICD suggesting decreased connectivity while conventional approaches suggest increased connectivity due to anesthesia. Seed connectivity for one of these regions (the left frontal lobe; green region) revealed both significant (p<0.05 corrected) increased and decreased connectivity, demonstrating that the different approaches may be sensitive to different aspects of changes in connectivity.

To further explore the changes in areas of *both* increased and decreased connectivity detected by coupled-ICD, a follow-up seed-based analysis was performed using the right parietal region (MNI coordinates: 51, −53, 30, volume = 3000 mm^3^). This analysis was performed on a set of images independent from the runs used to generate the coupled-ICD results. As expected from the coupled-ICD results, this seed region showed both significant (p<0.05, corrected) increased and decreased connectivity (Figure 4A). Increased connectivity was observed to the bilateral temporal lobes and to the left ventral orbitofrontal cortex. Decreased connectivity to the thalamus was also observed.

Both conventional voxel-based methods detected only regions of increased connectivity (Figure 3C and 3D). The coupled-ICD and matrix connectivity (Figure 3E) results suggest the presence of both increases and decreases in connectivity for the anesthesia condition. Additionally the results from the voxel-based methods suggests largely focal changes in connectivity; whereas, the coupled-ICD and matrix connectivity results are wide spread throughout the cortex.

While a general agreement was observed between all methods, for a region in the left frontal lobe/insular cortex, conventional approaches indicated an increase in connectivity while coupled-ICD indicated a decrease in connectivity for the anesthesia condition. We further investigated this region using a seed centered in the left frontal lobe (MNI: −33, 49, 13, volume = 1811 mm^3^). The seed analysis revealed that, during the anesthesia condition, this region showed increased connectivity to the bilateral parietal lobe and showed decreased connectivity to the thalamus (Figure 4B). This result suggests that, in some cases, conventional approaches and coupled-ICD may be sensitive to different aspects of changes in connectivity.

### Relaxing versus Drug-Related Imagery

**Figure 5:**
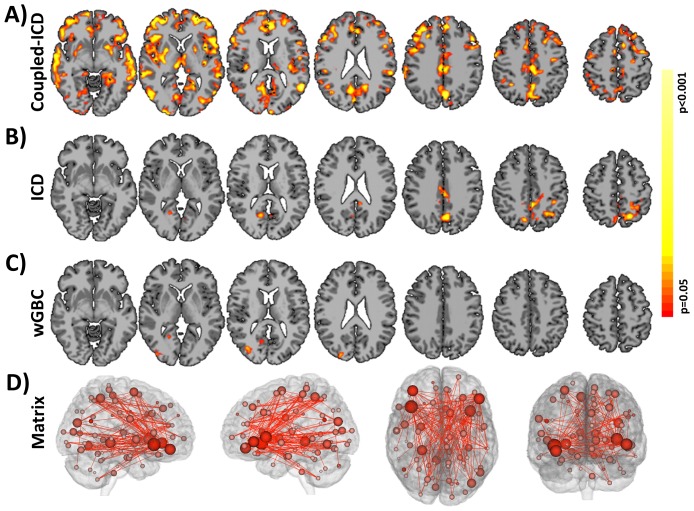
Comparison of coupled-ICD and conventional approaches for detecting group-by-condition interaction for cocaine-dependent subjects (CD) and healthy controls (HC). As the comparison between CD and HC subjects involves contrasting a metric (coupled-ICD) that already measures the difference between two conditions, this result can be interpreted in a similar manner to the interaction term of a classic 2×2 two-way ANOVA. A) Coupled-ICD detected more widespread significant interactions than the two conventional approaches, B) ICD and C) wGBC. D) ROI-based matrix connectivity method also detects widespread interaction between group and condition provide support that the coupled-ICD results are not simply artifacts. Only edges that were significantly difference at p<0.05 with FDR correction are shown. The size of the node is proportional to the number of significantly different edges touching that node. A larger node has more significantly different edges.

The performance of coupled-ICD was additionally explored with a second data set contrasting two groups of subjects—healthy controls (HC) and cocaine-dependent (CD) subjects—under paired acquisition conditions (relaxing or drug-related imagery). As the comparison between drug-dependent and control subjects involves contrasting a metric (coupled-ICD) that already measures the difference between two conditions, this result can be interpreted in a similar manner to the interaction term of a classic 2×2 two-way ANOVA. Coupled-ICD detected widespread significant (p<0.05, corrected) interactions between condition and group (Figure 5A). These regions included the PCC/precuneus, bilateral angular gyrus, bilateral insular cortex, bilateral putamen, SMA/ACC, and regions in the medial and lateral prefrontal cortex. The interactions detected by coupled-ICD were visually compared with interactions detected by the conventional voxel-based approaches (ICD and wGBC) and the matrix connectivity approach (Figure 5B-D). ICD detected significant interactions in the ACC and precuneus. WGBC detected significant interaction in the right lateral occipital lobe. Matrix connectivity detected widespread condition X group interaction with the largest effect being seen in lateral frontal regions. The regions detected by the conventional approaches (ICD, wGBC, and matrix connectivity) were also detected by coupled-ICD. The equivalent of simple main effects for each group is shown in supplemental material (Figure S7).

**Figure 6:**
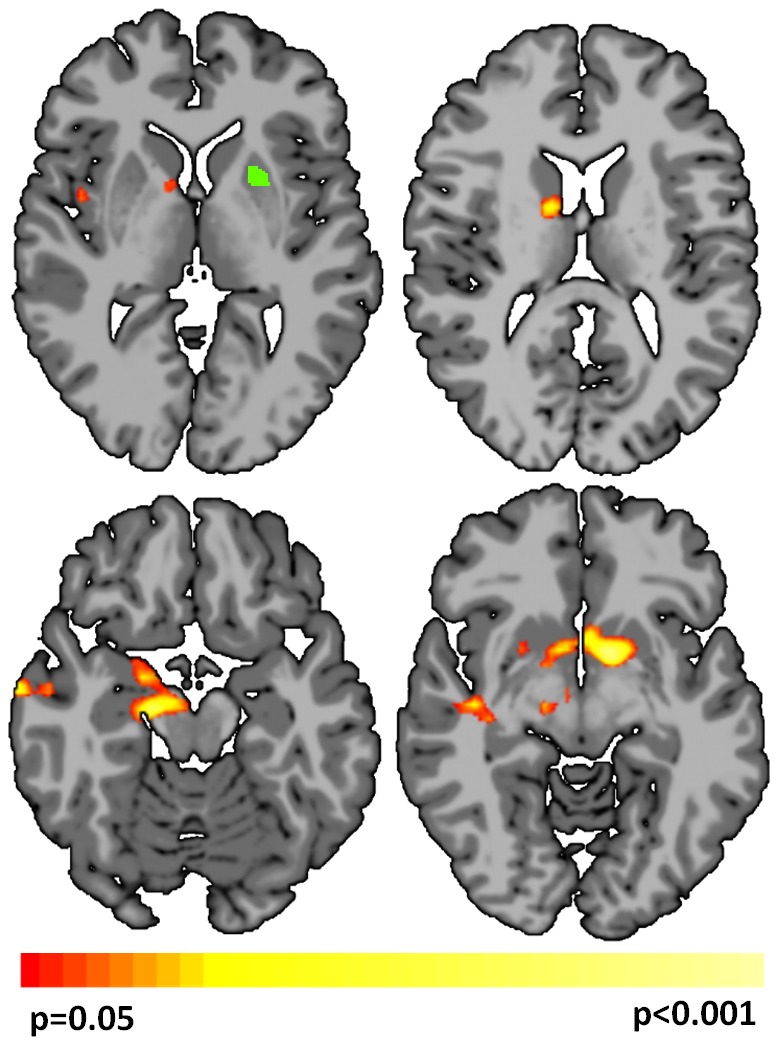
Follow-up seed-based connectivity results using a seed detected by coupled-ICD. A follow-up, seed-based connectivity analysis was performed using a region in the left putamen detected by coupled-ICD but not by conventional ICD and degree analysis. This region shows significant group interaction (p<0.05, corrected) in the caudate and nucleus accumbens. The subjects analyzed in the seed analysis were not used in the voxel-based analysis, providing additional evidence of coupled-ICD robustness and utility. The green region shows the location of the seed ROI.

To further investigate the differences detected by the coupled-ICD approach, a follow-up seed-based analysis was performed using a seed defined in the left putamen (MNI: −23, 10, −2, volume = 655 mm^3^) where significant between-group differences were found using coupled-ICD. The left putamen was chosen as a seed region due to the substantial literature (e.g., [25] [15]) implicating this region in drug addiction. Significant (p<0.05, corrected) interactions between group and condition were observed in the bilateral caudate and nucleus accumbens (Figure 6).

## Discussion

We present a novel method, coupled-ICD, for analyzing differences in functional connectivity at the voxel level for paired data. Current approaches to voxel-based measures of functional connectivity generally compress information about the connections to a voxel into a few summary statistics. Applying conventional methods to data with paired conditions where each condition is summarized separately can be suboptimal, as separately summarizing each condition of a pair can mischaracterize important changes in connectivity (see Figure 1). Thus, in order to maximize the detection power of connectivity in resting-state fMRI (rs-fMRI), each condition should not be analyzed separately. Our solution, coupled-ICD, jointly analyzes each condition by modeling the difference of the connectivity patterns of two paired conditions for a subject. We show that coupled-ICD can detect additional regions of difference between two paired conditions. Standard seed-based analyses performed on data independent from the coupled-ICD results show that the regions detected by coupled-ICD show significant differences in connectivity.

Voxel-based metrics can be used as a data-driven way to define seed regions for ROI-based analysis [6], [23], [26]. However, voxel-based approaches and ROI-based approaches can often produce seemingly conflicting results—for example, voxel-based results may suggest an increase in connectivity for a region while follow-up seed analysis with the region may show decreases in connectivity to the region. This discrepancy arises because the two approaches are fundamentally different. In the ROI-based approach, paired information about which connections have changed is preserved as each connection is directly compared to the same connection under different conditions. In previous voxel-based approaches, this paired information is lost. Because conventional voxel-based approaches compare summary metrics of the connections, only the overall strength of connections to any given voxel is compared. Coupled-ICD differs from other voxel-based approaches in that it directly compares each connection under two conditions (similar to ROI-based approaches) and then summarizes these differences. This approach provides a direct link between coupled-ICD and any subsequent ROI-based follow-up analyses. This direct link does not always exist with conventional methods that inherently compare nodes instead of edges.

Voxel-based approaches to rs-fMRI can be problematic due to the large number of nodes and edges that must be kept in memory. Thus, most studies when presented with paired data analyze each condition separately. While approaches for comparing edges using ROI-level graphs have been developed [19], [27], [28], graphs at the voxel level become difficult to analyze due to memory constraints and multiple comparison issues with groups of 10 or more subjects. However, for the special case of paired scans, only two graphs need to be analyzed simultaneously, reducing the complexity of this problem. This simplification allows coupled-ICD to mimic these ROI-based approaches and compare differences in edges themselves, not differences in a summary statistic of edges. Thus, with the coupled-ICD approach, edges that increase in connectivity and edges that decrease in connectivity can be analyzed separately allowing for the detections of regions showing increased *and* decreased connectivity (as shown in data set 1). The results from the coupled-ICD approach more closely resemble the matrix connectivity (ROI-to-ROI) results than the conventional voxel-based results, further highlighting the similarity between coupled-ICD and ROI approaches.

Coupled-ICD's main value is as a first-pass, screening method to identify brain regions that could be important for follow-up seed-to-whole brain analyses and/or used to test specific hypotheses. As coupled-ICD is an exploratory screening method, increased sensitivity is more essential than increased specificity. Even though seed-to-whole brain connectivity remains the primary method to test for changes in connectivity to a specific region, only a few, select regions, based on a priori information, can be tested with adequate power. If a region is not identified in an exploratory analysis, important and novel findings may be missed. This could create a bias in the literature where subtle, but complex changes are under reported (Lieberman and Cunningham, 2009). Thus, exploratory methods, such as coupled-ICD, provide value to screen for potential seed regions and can be used for further analysis.

Our new coupled-ICD approach can be used to better quantify differences between paired conditions, which we demonstrate using two data sets. For the first data set, comparisons between subjects under awake and anesthetized conditions using coupled-ICD revealed widespread differences in the medial frontal lobe, SMA, ACC, thalamus, and visual cortex (see Figure 3). Many of these regions are consistent with previous analysis of this data [5], [13]. However, neither of these previous analyses detected the connectivity changes in the lateral frontal lobe that were revealed coupled-ICD, an approach specifically designed for analyzing paired data. This region was not *a priori* hypothesized to show changes under anesthesia and thus was not examined with ROI-based approaches [13]. Similarly, this region was not detected by conventional *degree* analysis [5]. However, the coupled-ICD approach showed strong evidence of large connectivity differences for this regions.

For the second data set, we found significant differences in the PCC, bilateral angular gyrus, bilateral insular cortex, bilateral putamen, medial prefrontal/ACC, and visual-processing areas (Figure 5). These findings support many previous studies implicating the putamen [15], [29], insula [15], [25], ACC [15], [30], and prefrontal regions [29] in cocaine addiction. An example follow-up seed-based analysis using a seed in the left putamen revealed that the coupled-ICD differences in the left putamen are mostly due to differences in connectivity to the limbic lobe (Figure 6). Together, the coupled-ICD results and the follow-up seed analysis suggest a disruption of corticostriatal-limbic connectivity in the presence of drug cues for cocaine-dependent subjects.

Although coupled-ICD gains additional paired information by jointly analyzing two conditions, it is currently limited to the special case of only two paired conditions. In experiments that do not use paired conditions, conventional approaches such as ICD remain the more appropriate choice. Further, even if more than two paired conditions are used, coupled-ICD can only jointly analyze two conditions at a time. Future work could involve generalizing coupled-ICD to any number of conditions. Finally, while there is no significant difference in motion between groups for either data set, confounds related to head movement may still exist.

Numerous clinical applications could benefit from measuring changes in the functional organization of the brain at the voxel level, yet the translational technology for detecting changes in connectivity remains elusive. We present a method for exploratory analysis of paired conditions to detect regions that differ in their connectivity patterns between conditions. This method, coupled-ICD, jointly examines the connections to a voxel across two conditions instead of investigating each condition separately as in conventional voxel-based network approaches. Using two data sets, we show that coupled-ICD detected differences between paired conditions that were not observed with conventional approaches, suggesting that conventional approaches can underestimate the differences between paired conditions. Using independent data, follow-up seed-based analyses using these regions as seed ROIs provided additional evidence that these regions exhibited changes in connectivity as a function of experimental condition. Consequently, coupled-ICD has promise as a method for assessing changes in functional connectivity pre- and post-treatment and fills an important void not currently covered by conventional approaches.

## Supporting Information

Figure S1 Original results for coupled-ICD increases in the anesthesia data set presented with additional slices.
(TIF)

Figure S2 Replication results for coupled-ICD increases in the anesthesia data set presented with additional slices.
(TIF)

Figure S3 Original results for coupled-ICD decreases in the anesthesia data set presented with additional slices.
(TIF)

Figure S4 Replication results for coupled-ICD decreases in the anesthesia data set presented with additional slices.
(TIF)

Figure S5 Original results for coupled-ICD in the cocaine-dependence data set presented with additional slices (p<0.05, corrected).
(TIF)

Figure S6 Replication results for coupled-ICD in the cocaine-dependence data set presented with additional slices (p<0.05, corrected).
(TIF)

Figure S7 Simple main effect for relaxing versus drug related imagery. The simple main effects of condition for the cocaine dependent subjects and healthy controls are shown for A) coupled-ICD, B) ICD, and C) wGBC. All methods indicate a larger change in connectivity for the dependent subject due to condition than for the control subjects. These changes in the dependent subjects are likely responsible for the significant interactions in the main text.
(TIF)
