# Supplementary figures and images for: Coupled Intrinsic Connectivity Distribution Analysis: A Method for Exploratory Connectivity Analysis of Paired fMRI Data
Source: PLoS One. 2014 Mar 27;9(3):e93544. doi: 10.1371/journal.pone.0093544 (PMC3968179; doi:10.1371/journal.pone.0093544)

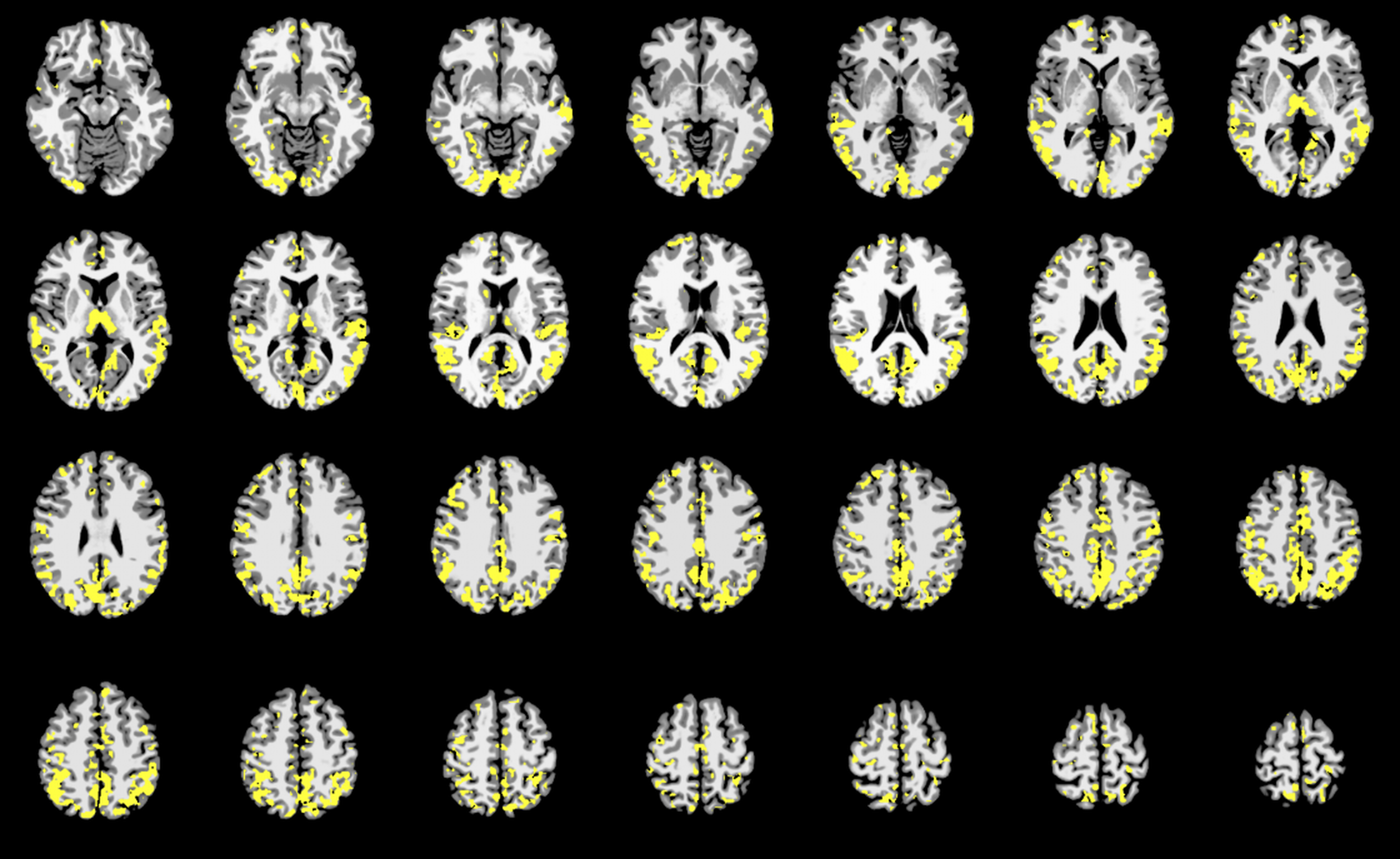

Supplement: Figure S1 — Original results for coupled-ICD increases in the anesthesia data set presented with additional slices. (TIF) [file pone.0093544.s001.tif]

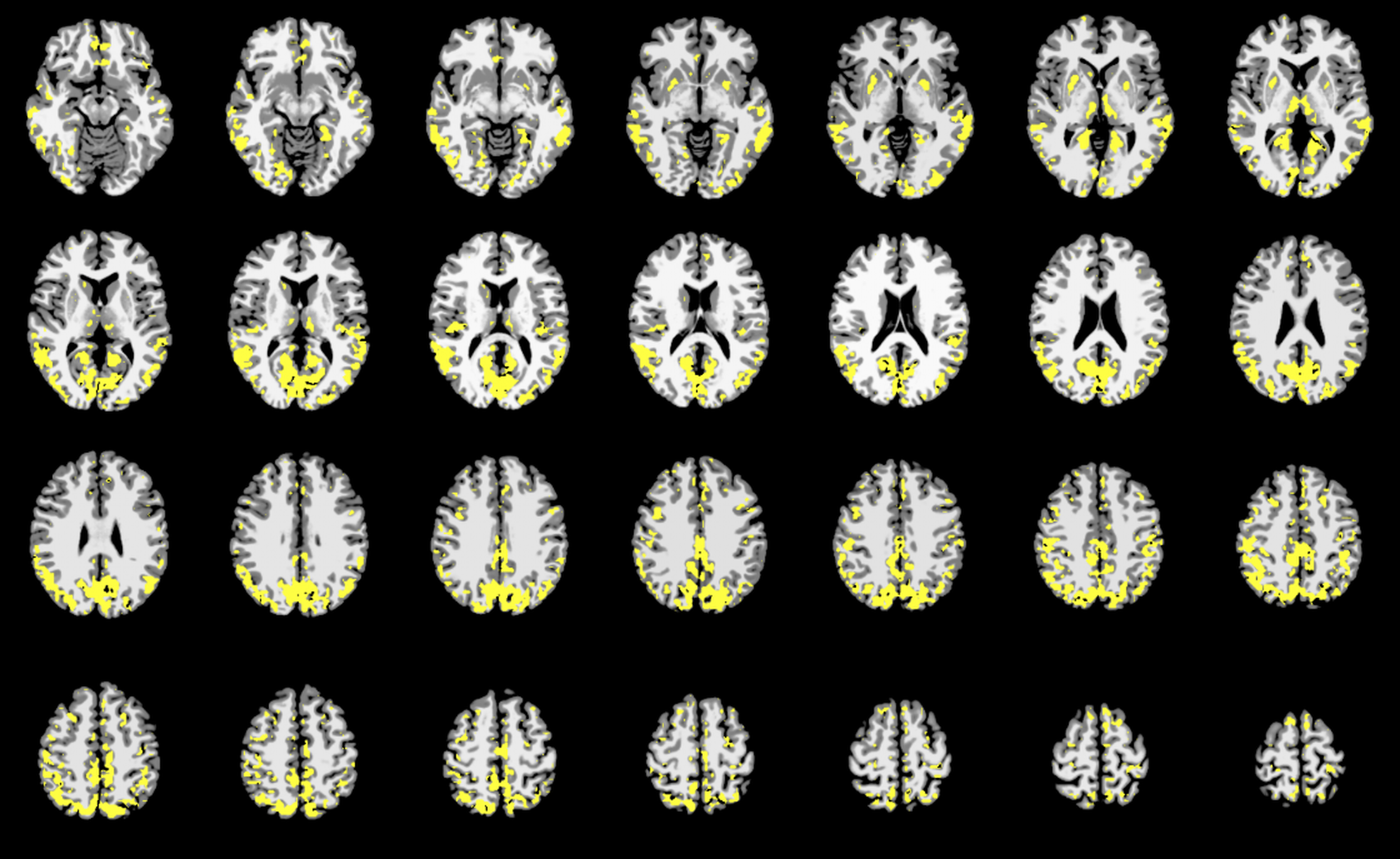

Supplement: Figure S2 — Replication results for coupled-ICD increases in the anesthesia data set presented with additional slices. (TIF) [file pone.0093544.s002.tif]

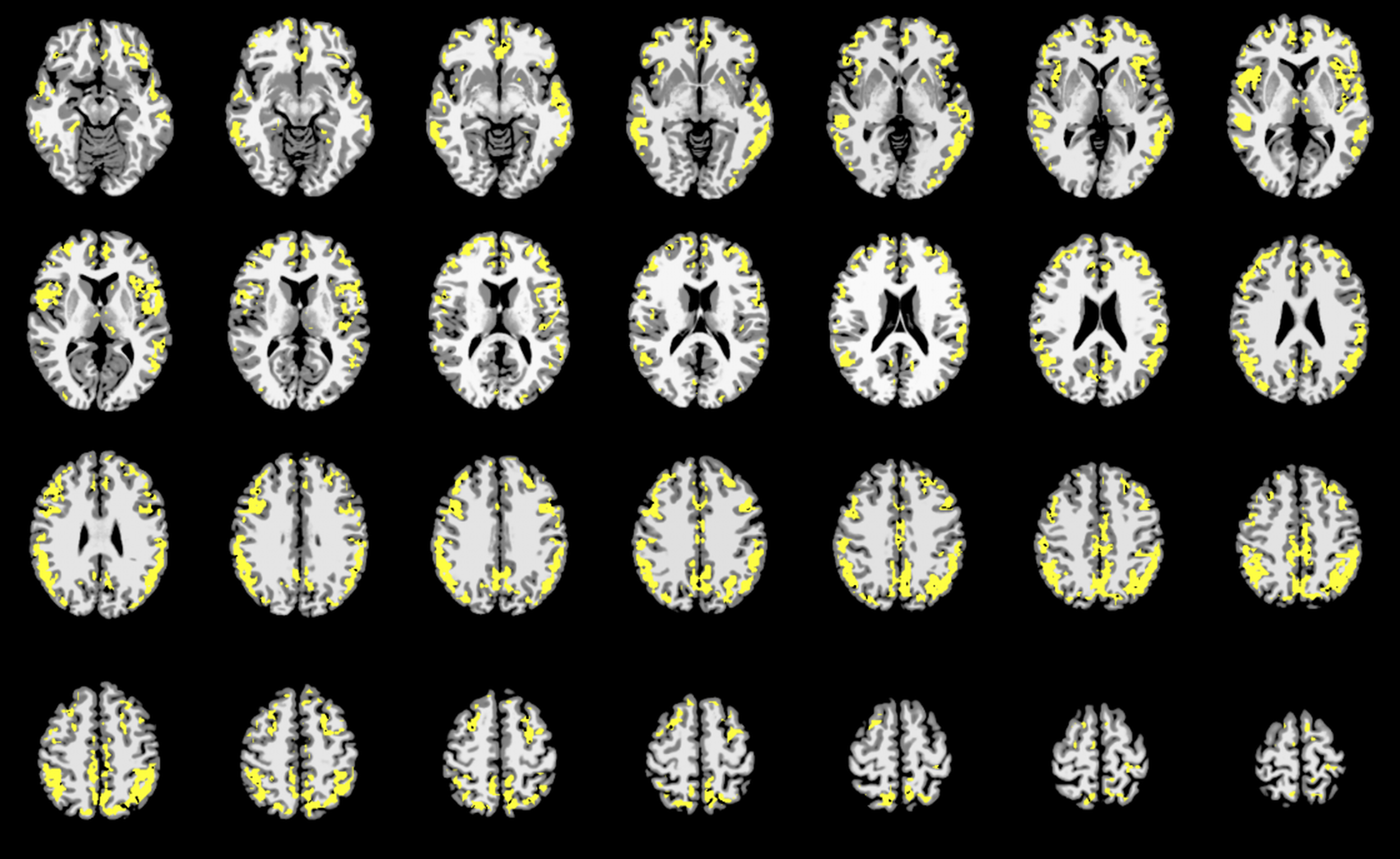

Supplement: Figure S3 — Original results for coupled-ICD decreases in the anesthesia data set presented with additional slices. (TIF) [file pone.0093544.s003.tif]

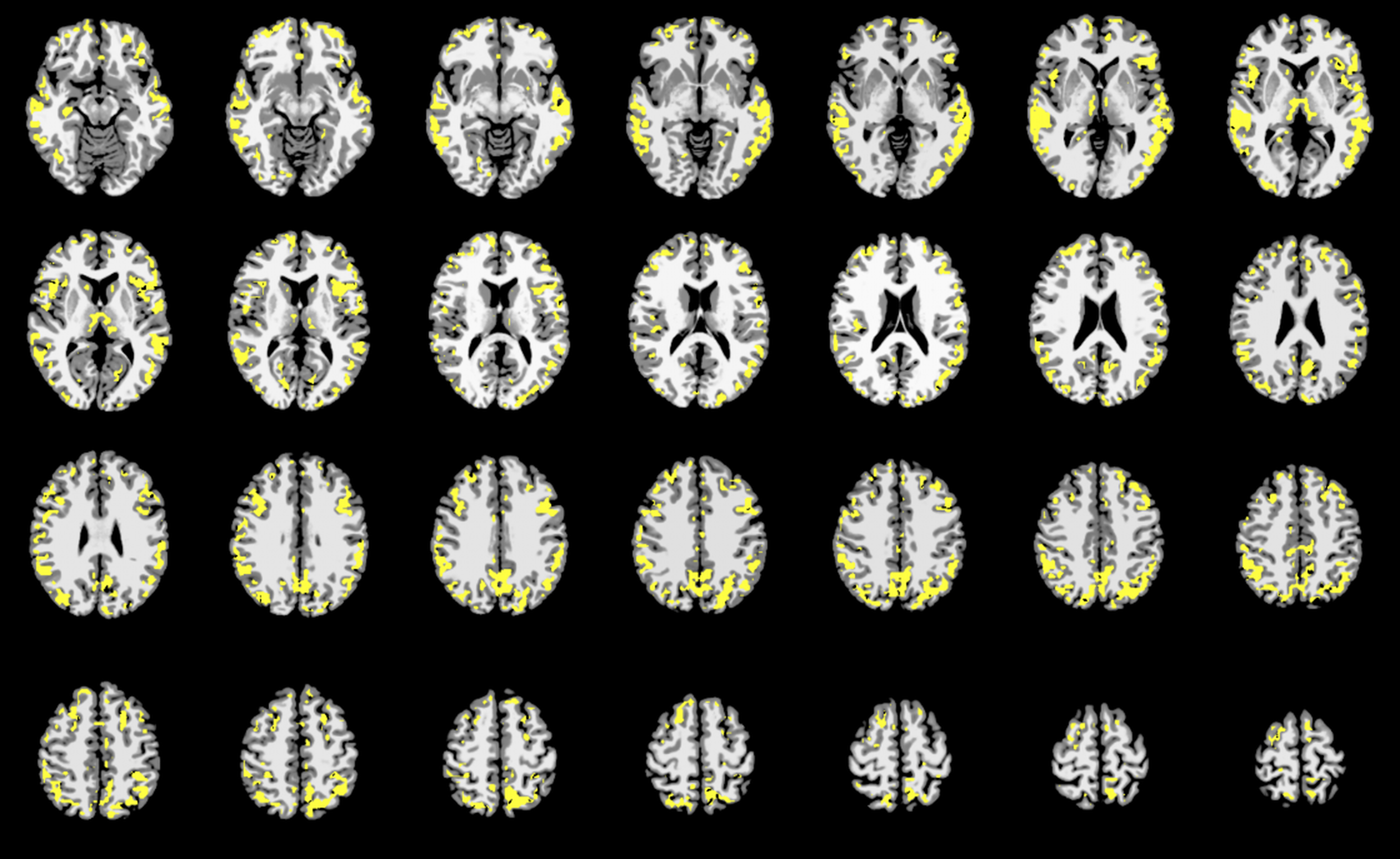

Supplement: Figure S4 — Replication results for coupled-ICD decreases in the anesthesia data set presented with additional slices. (TIF) [file pone.0093544.s004.tif]

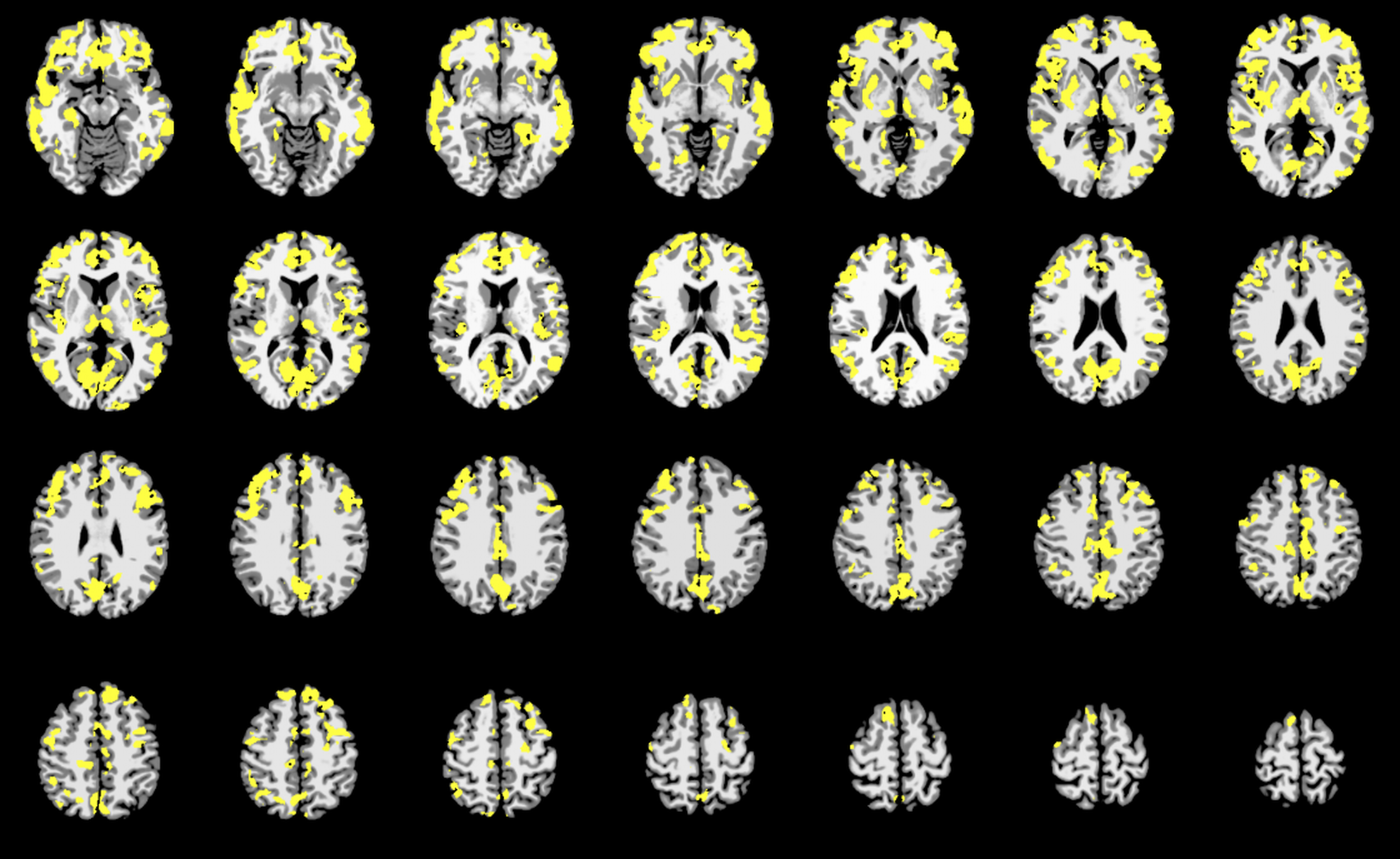

Supplement: Figure S5 — Original results for coupled-ICD in the cocaine-dependence data set presented with additional slices (p<0.05, corrected). (TIF) [file pone.0093544.s005.tif]

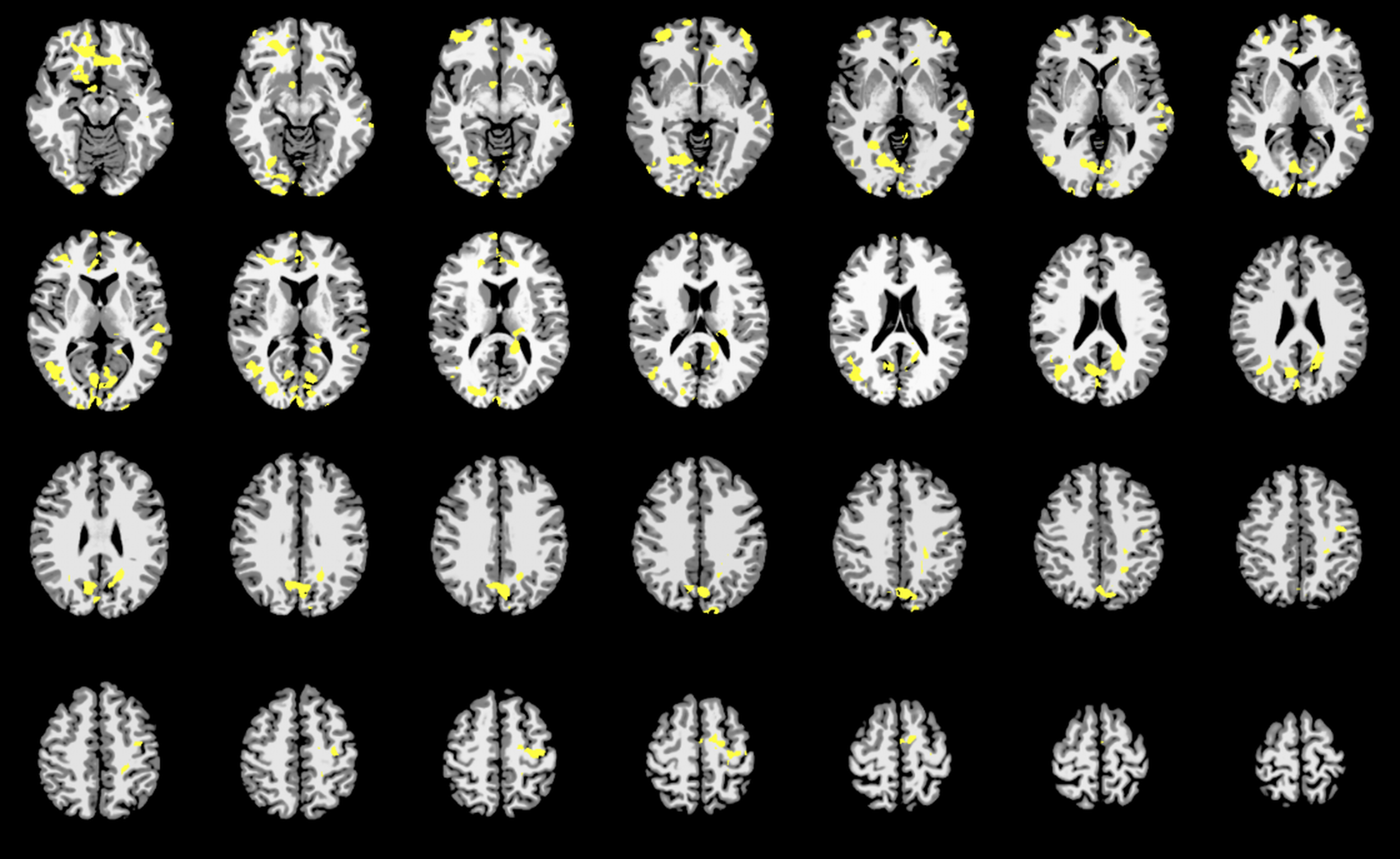

Supplement: Figure S6 — Replication results for coupled-ICD in the cocaine-dependence data set presented with additional slices (p<0.05, corrected). (TIF) [file pone.0093544.s006.tif]

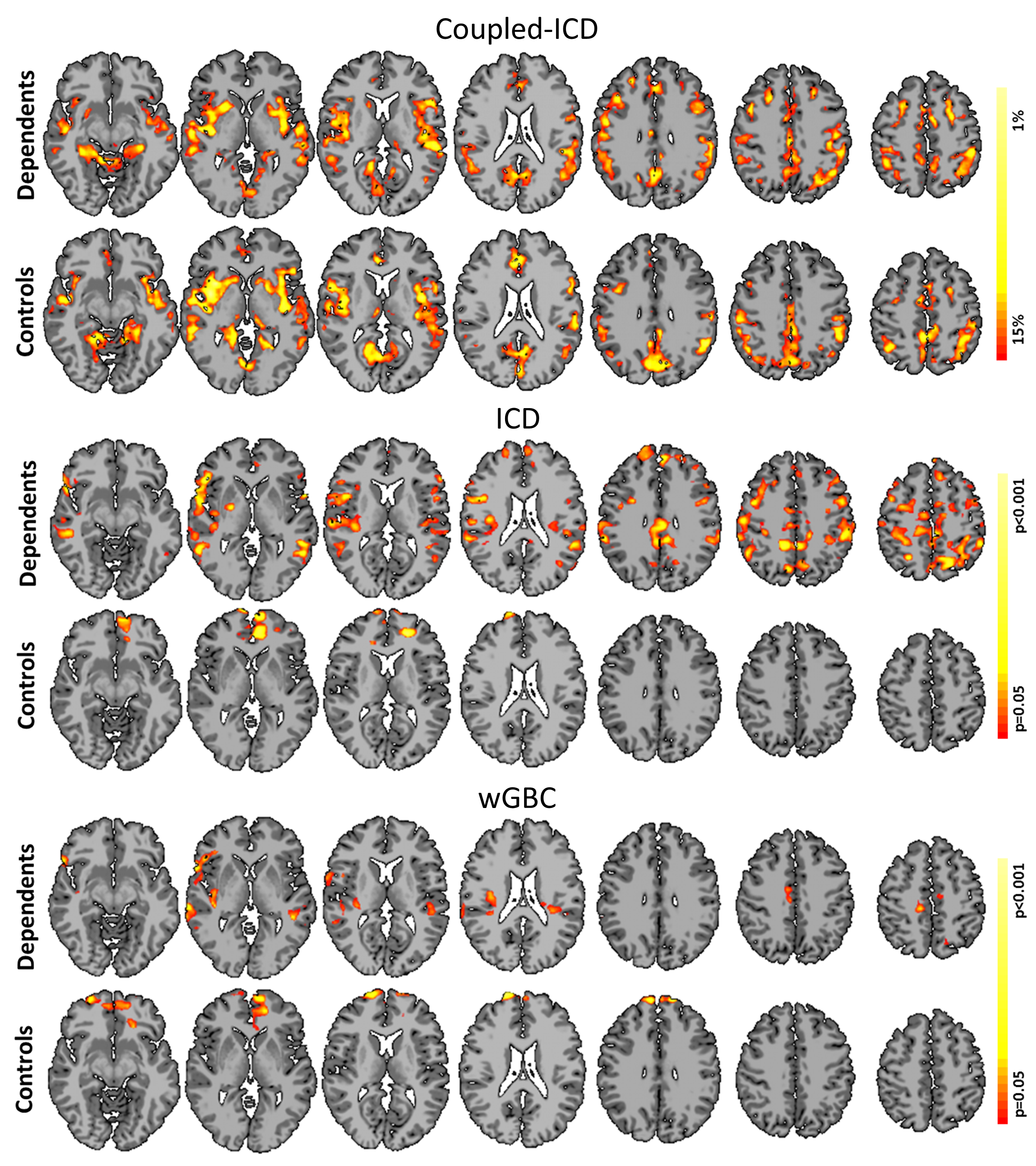

Supplement: Figure S7 — Simple main effect for relaxing versus drug related imagery. The simple main effects of condition for the cocaine dependent subjects and healthy controls are shown for A) coupled-ICD, B) ICD, and C) wGBC. All methods indicate a larger change in connectivity for the dependent subject due to condition than for the control subjects. These changes in the dependent subjects are likely responsible for the significant interactions in the main text. (TIF) [file pone.0093544.s007.tif]
